# Effect of Different High-Fat and Advanced Glycation End-Products Diets in Obesity and Diabetes-Prone C57BL/6 Mice on Sperm Function

**DOI:** 10.22074/IJFS.2021.137231.1022

**Published:** 2021-06-22

**Authors:** Fahimeh Akbarian, Mohsen Rahmani, Marziyeh Tavalaee, Navid Abedpoor, Mozhdeh Taki, Kamran Ghaedi, Mohammad Hossein Nasr-Esfahani

**Affiliations:** 1 Department of Animal Biotechnology, Reproductive Biomedicine Research Center, Royan Institute for Biotechnology, ACECR, Isfahan, Iran; 2 Department of Animal Biotechnology, Cell Science Research Center, Royan Institute for Biotechnology, ACECR, Isfahan, Iran

**Keywords:** Advanced Glycosylation End Products, Diabetes Mellitus, High-Fat Diet, Reactive Oxygen Species, Sperm Parameters

## Abstract

**Background:**

We aimed to compare the effects of using high-fat (HF) and advanced glycation end-products (AGEs) containing diets to induce obesity and diabetes on sperm function in mice.

**Materials and Methods:**

In this experimental study, twenty-five 4-week old C57BL/6 mice were divided into 5 groups and were fed with control, 45% HF, 60% HF, 45% AGEs-HF, or 60% AGEs-HF diet. After 28 weeks, fast blood sugar, glucose intolerance, insulin concentration, homeostatic model assessments (HOMA) for insulin resistance (IR) and HOMA for beta cells (HOMA beta) from systematic blood were assessed. In addition, body weight, morphometric characteristics of testes, sperm parameters, DNA damage (AO), protamine deficiency (CMAA3), and sperm membrane (DCFH-DA) and intracellular (BODIPY) lipid peroxidation were measured.

**Results:**

Body mass and fasting blood sugar increased significantly in all experimental groups compared to the control group. Insulin concentration, glucose intolerance, HOMA IR, and HOMA beta were also increased significantly with higher levels of fat and AGEs in all four diets (P<0.05). The changes in the 60% HF-AGEs group, however, were more significant (P<0.001). Morphometric characteristics of the testis, sperm concentration, and sperm morphology in the diet groups did not significantly differ from the control group, while sperm motility and DNA damage in the 45%HF were significantly low. Although for protamine deficiency, both 60% HF-AGEs and 45% HF showed a significant increase compared to the control, the mean of sperm lipid in the 45% HF group and intracellular peroxidation in the 60% HF-AGEs group had the highest and the lowest increases, respectively.

**Conclusion:**

Our results, interestingly, showed that is the negative effects of a diet containing AGEs on examined parameters are less than those in HF diets. One possible reason is detoxification through the activation of the protective glyoxalase pathway as the result of the chronic AGEs increase in the body.

## Introduction

Diabetes is a complex chronic condition that results in high levels of blood sugar and is one of the main causes of disability and death worldwide. It is mainly caused by either insulin deficiency due to the destruction of insulin-producing beta cells of the pancreas (Fig Type 1 diabetes), or insensitivity of cells to insulin (Type 2 diabetes) (1). Developing type 2 diabetes is strongly associated with obesity, as 58% of global diabetes cases are attributed to body mass index higher than 25 kg/m^2^ based on the World Health Organization (WHO) reports (2). As the global trends in diabetes are alarming, WHO has recommended the promotion of healthy diets and physical activity in societies as an attempt to reduce obesity and diabetes as well as to manage and lower their complications. 

Mounting evidence highlights the close relationship between the adoption of western dietary pattern and the steady increase in worldwide obesity and diabetes over the past few decades (1). The western diet is characterized by high consumption of refined sugars and saturated fat, but insufficient amounts of fiber (3). This diet is rich in advanced glycosylation end-products (AGEs), which are highly reactive molecules produced by a non-enzymatic reaction known as the Maillard reaction between reducing sugars, such as glucose and other compounds such as proteins, nucleic acids, or lipids. Intra- and extracellular accumulation of AGEs interfere with various proteins and several cellular functions (4).

Although endogenous AGEs are constantly produced in the body during the glycation of various biomolecules, they can also be originated from exogenous sources in the process of modern methods of preparing precooked meals heated in high temperatures (3). In this context, it has been found that around 10% of exogenous AGEs that are taken up are eliminated by the body and the rest lead to increased AGE serum levels and elevated fat deposits in various tissues (4). Several conditions including hyperglycemia and insulin resistance (IR) accelerate the formation of AGEs so that the intracellular levels of these compounds could elevate 14-fold faster in a high glucose state (5). AGEs are considered as the main pathogenic factors in the development and progression of complications associated with diabetes (3). The production of reactive oxygen species (ROS) by AGEs is one of the biochemical mechanisms of diabetes pathology (4).

Physiological alterations driven in part by diabetes mellitus could affect the functions of male reproduction through several mechanisms and pathways as it disturbs the endocrine regulation of steroidogenesis, spermatogenesis, sperm maturation, as well as of penile erection and ejaculation. Proper glucose metabolism plays a pivotal role in spermatogenesis and fertilization capacity of mature sperm, as well as maintenance of basic cells (6). Fertility problems associated with obesity and diabetes mainly arise from unbalanced ROS and subsequent oxidative stress. ROS encompasses a vast number of detrimental effects on male reproductive functions. This is supported by the findings that showed antioxidant therapy to be beneficial for improving the sperm parameters in diabetic men (7).

Although the adverse consequences of diets rich in saturated fat and AGEs compounds on male fertility have been suggested in previous studies (4-7), there are some discrepancies in the literature and also there is no study that has simultaneously assessed the effects of high-fat (HF) and AGEs-containing diets on sperm function. In this context, the current study was conducted to compare the effects of obesity and diabetes in C57BL/6 male mice fed with diets containing different levels of saturated fat and AGEs compounds, to better understand their effects on infertility, by analyzing the sperm parameters, oxidative stress, and chromatin status, and also to investigate the mechanisms through which these diets could induce infertility.

## Materials and Methods

### Design of experiment

This experimental study was approved by the institutional review board from the Royan Institute (No: 97000269) and performed under the supervision of the animal Ethics Committee of Royan Institute. Twentyfive 4-weeks old healthy non-obese and non-diabetic C57BL/6 male mice were selected from Institute for Biotechnology (Isfahan, Iran) and housed under controlled conditions; temperature of 21 °C (± 2%), 65% humidity (± 5%), 12‐hours light/12‐hours dark cycles, and an ad libitum access to food and water. After one week of acclimatization in special cages, mice were randomly divided into five groups (control/ chow diet, 45%, and 60% HF diet groups, 45% and 60% AGEs diet groups; for each group, n=5 were considered based on the Kolmogorov-Smirnov test. The mice received the experimental diets when they were 5 weeks old. After 28 weeks of feeding special diets for inducing obesity and diabetes (8-10), body mass, fasting blood sugar, insulin concentration, glucose intolerance, homeostatic assessment of insulin resistance (HOMA IR) and HOMA for beta cells (HOMA beta) along with the weight and morphometric characteristics of testes (width, length, and thickness of the left and right testis), sperm parameters (concentration, motility, abnormality), and sperm function (protamine deficiency, DNA damages, membrane lipid and intracellular peroxidation) were measured in each group of mice.

### Diets

The HF diet and AGE were obtained from Royan Biotechnologist immune-company (Iran, Tehran). Preparing the formulation of the diets was based on previous studies (11). Four types of diets were applied in this study. 45% HF and 60% HF groups, which 45% and 60% of the calories were provided from lipids respectively, as well as 45% HF- AGEs and 60% HF- AGEs, in which lipids and AGEs provide the 45% and 60% of the calories for each group. Notably, the fat ingredients of the diets are saturated. The details of the composition of the five diet groups are presented in Table 1. This study is the continuation of the study by Abedpoor et al. (unpublished data), and the results of several factors such as glucose tolerance test, fasting blood sugar, insulin concentration, HOMA-IR, and HOMA-beta are similar between these two studies.

**Table 1:** Characteristics of special mouse diets for each different studied group


Diet composition (% w/w)	Diet groups (n=5)				
		Normal diets	45% HF	60% HF	45% HF-AGEs	60% HF-AGEs

Protein	20.56	19.4	20	28	23
Fat	12.55	45	60	45	60
Carbohydrate	47.71	21.59	13.8	15.49	9.56
Fiber	3.8	2.26	1.2	3.35	0.96
Ash	10.38	7.85	0.9	7.18	5.9
Mois‌ture	5	3.9	4.1	0.98	0.58
Calories (kcal/g)	3.8	5.6	6.7	5.8	6.7


HF; High-fat diet and AGEs; Advanced glycation end-products.

### Fasting blood sugar and glucose tolerance test

Fasting blood sugar was measured from the tail vein by animal glucometer after 6 hours of fasting, following 28 weeks of keeping the mice on the special diets. Tolerance test was also performed after 28 weeks of keeping the mice on the special diets. After 6 hours of fasting, D-glucose (solution of 10 g/dL at a dose of 1 g/kg and volume load of 10 mL/g body mass, Sigma, Australia) was injected intraperitoneally (12, 13) and glucose level was measured at 15, 30, 60, and 90 minutes intervals from the tail vein. Blood glucose concentration was measured during daytime-fasting (14), using an animal glucometer (Alpha TRAK). The mice were sacrificed following these evaluations. 

### Insulin concentration

The mice were euthanized under combined administration of xylazine (10 mg/kg body mass per mouse) and ketamine (80 mg/kg body mass per mouse). For assessing the insulin concentration, heart blood was collected immediately after scarification. Following centrifugation of the collected blood at 4500 rpm for 5 minutes at 4°C, the insulin level in the serum (ng/dL) was quantified using Ultra-Sensitive Mouse Insulin ELISA kit (Crystal Chem, USA) according to the manufacturer’s instructions.

### Homeostatic model assessment of insulin resistance

HOMA IR, a surrogate marker of IR was assessed using fasting glucose and insulin concentration according to the following formula (15):

equation(1)

### Homeostatic model assessment of beta cells

HOMA-beta assesses the function of β-cells using insulin concentration according to the following formula (16):

equation(2)

### Sperm collection

Following the sacrifice of mice, weight and morphometric measurements of the testes (width, length, and thickness of the left and right testis) were performed. The cauda segment was separated from the left epididymis and cut into pieces and incubated in 2 ml of sperm washing media+10% serum at 37°C for 30 minutes to retrieve spermatozoa. After analyzing the sperm parameters, the spermatozoa were washed with phosphate-buffered saline (PBS, Sigma, Australia) for further evaluation.

### Assessment of sperm parameters 

Sperm concentration (million/ml) and sperm motility (% motile) were measured using a sperm counting chamber (Sperm meter, Sperm Processor, India) under a light microscope. The collected sperm was stained by the Eosin-Nigrosin method as described previously for morphological evaluations (17). Abnormalities in the head, neck, and tail of the spermatozoa were assessed, and data were reported as the percentage of sperm abnormal morphology.

### Assessment of sperm protamine deficiency

Histone replacement by protamine occurs during the late stages of spermatogenesis. We evaluated the protamine deficiency using the chromomycin A3 (CMA3) staining method as described previously (17). For this evaluation, 200 spermatozoa were assessed under an Olympus fluorescent microscope (BX51, Japan) and protamine‐deficient spermatozoa (bright yellow or CMA3 positive) were distinguished from normal protamine content (dull yellow spermatozoa or CMA3 negative), and the percentage of protamine deficiency was reported for each sample. 

### Assessment of sperm DNA damage

The DNA damage was evaluated by acridine orange (AO) staining, as described previously (17). For this analysis, 200 spermatozoa were assessed by fluorescent microscope (BX51, Japan) and the percentages of spermatozoa with normal double-stranded DNA (green stained) and abnormal spermatozoa with denatured DNA (orange/ red stained) were calculated for each sample.

### Assessment of sperm membrane lipid peroxidation

Sperm membrane lipid peroxidation was assessed using the BODIPY probe as described previously (18). Briefly, BODIPY 581/591 C11 (D3861, Molecular Probes) with a concentration of 5 mM was added to 2×10^6^ spermatozoa and incubated at 37°C for 30 minutes and the percentage of lipid peroxidation was assessed using the FACSCalibur flow cytometer (Becton Dickinson, USA). A positive control for each sample was obtained by adding H_2_ O_2_ to sperm suspensions.

### Assessment of sperm intracellular reactive oxygen species

DCFH-DA staining was used to detect cytosolic ROS and peroxidation as described previously (19). Briefly, 106 spermatozoa were incubated with 0.5 μM DCFH-DA at 37°C for 30 minutes. Intracellular ROS was assessed using the FACSCalibur flow cytometer (Becton Dickinson, USA). A positive control for each sample was obtained by adding H_2_ O_2_ to sperm suspensions.

### Statistical analysis

All data in the present study were analyzed by the Statistical Package for the Social Sciences for Windows, version 25 (SPSS, Inc., Chicago, IL, USA). All the parameters had a normal distribution, and a one‐way analysis of variance (ANOVA) was used to compare the sperm parameters, lipid peroxidation, and chromatin status. Data were presented as mean ± standard error of the mean, and P<0.05 was considered significant.

## Results

### Effects of different diets on body mass, weight, and morphometric characteristics of testes

The initial and final mean body mass of the mice in the five studied groups is presented in Table 2. All the groups gained weight significantly compared to the control group after 28 weeks of being fed with the special diets. In addition, we assessed morphometric characteristics of testes (width, length, and thickness of the left and right testis) and found that none of them showed any significant differences in the mean values compared to their corresponding control group. On the other hand, unlike the 45% HF group (0.101 ± 0.001, P>0.05), the mean weight of the left testis in the 60% HF (0.107 ± 0.003, P<0.05), 45% HF-AGEs (0.116 ± 0.009, P=0.001), and 60% HF-AGEs (0.120 ± 0.003, P<0.001) groups significantly increased in comparison to the control group (0.084 ± 0.004). Additionally, the mean weight of the left testis in the 60% HF-AGEs group increased compared to the 45% HF group (P<0.05).

**Table 2:** Body weight of different studied groups at the beginning of the study and after 28 weeks of feeding special diets


Groups (n=5)	Body weight (g)		
		Baseline	After 28 weeks	Weight gain

Control	14 ± 0.02	26 ± 0.5	12 ± 0.3
45% HF	13 ± 1.5	50.5 ± 0.5^*^	37 ± 0.4^*^
60% HF	14.1 ± 1	37.8 ± 0.2^*^	23 ± 0.1^*^
45% HF-AGEs	12 ± 2	43 ± 1^*^	31 ± 0.8^*^
60% HF-AGEs	13.5 ± 1.5	62 ± 0.5^*^	48.5 ± 1^*^


Data are expressed as means ± standard error of the mean. HF; High-fat diet, and AGEs; Advanced glycation end-products. Significant difference is presented as *P<0.05.

### Effects of different diets on glucose level and insulin status

Glucose tolerance was dropped along with the increase in fat and AGEs content in the diet of the different studied group, so that the 60% HF-AGEs was the most intolerant group to glucose compared to the control group (P<0.001). The results of fasting blood sugar showed a significantly increased level of this parameter in all four groups with a special diet in comparison to the control (P<0.05).

As shown in Table 3, the results of insulin concentration, HOMA-IR, and HOMA-beta were significantly higher in all groups with a special diet compared to the control group (P<0.05 for 45% HF, 60% HF, 45% HF-AGEs and P<0.001 for 60% HF-AGEs group). Fasting blood sugar insulin concentration and IR were higher in 60% HF-AGE compared to the other groups. Therefore, we considered 60% HF-AGE and 60% HF groups as type 2 diabetes, and pre-diabetes groups, respectively.

**Table 3:** Insulin concentration and homeostatic model assessment (HOMA) in different studied groups after 28 weeks of keeping C57/BL6 mice on special diets


Groups (n=5)	Insulin concentration (ng/mL)	HOMA-insulin resis‌tance	HOMA-beta

Control	0.35 ± 0.05	0.09 ± 0.01	3.06 ± 0.25
45% HF	1.29 ± 0.04^*^	0.63 ± 0.02^*^	4.31 ± 0.21^*^
60% HF	0.82 ± 0.09^*^	0.31 ± 0.02^*^	3.15 ± 0.20
45% HF-AGEs	1.62 ± 0.07^*^	0.78 ± 0.01^*^	5.27 ± 0.27^*^
60% HF-AGEs	3.95 ± 0.19^*^^*^	2.56 ± 0.13^*^^*^	7.15 ± 0.28^*^^*^


Data are expressed as means ± standard error of the mean. HF; High-fat diet, AGEs; Advanced glycation end-products. Significant difference is presented as *P<0.05 and **P<0.01.

### Effects of different diets on conventional sperm parameters

**Fig.1:**
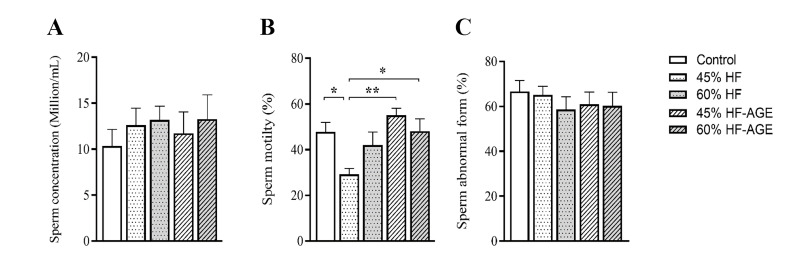
Sperm parameters of different studied groups after 28 weeks of feeding C57/BL6 mice with special diets. **A.** Sperm concentration (10^6^ /mL). **B.** Sperm motility (%). **C.** Total sperms with abnormal morphology (%). Data are expressed as means ± standard error of the mean. HF; High-fat diet, AGEs; Advanced glycation end-products, * ; P<0.05, and **; P<0.01.

Conventional sperm parameters are demonstrated as bar charts in Figure 1. The mean sperm concentration (10^6^ /ml), the mean percentage of sperms with total abnormal morphology as well as abnormal head and tail were not significantly affected by HF and HF-AGEs diets for 28 weeks. However, the mean motility of the sperms in the 45% HF group decreased compared to the control (P<0.05), whilst the mean motility of the sperms in the 45% HF-AGEs (P=0.004) and 60% HF-AGEs (P<0.05) groups increased compared to the 45% HF group.

**Fig.2:**
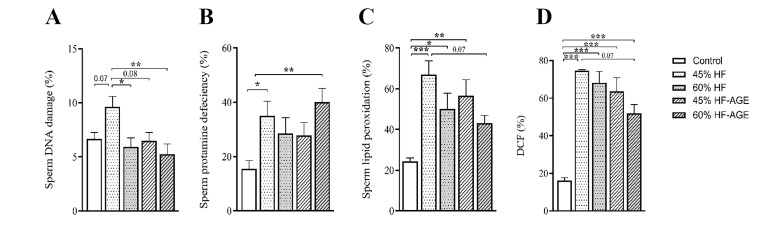
Comparison of sperm function tests within groups. **A.** Sperm DNA damages as shown by acridine orange staining (%) and **B.** Sperm protamine deficiency indicated by chromomycin A3 staining (%) in different study groups after 28 weeks of feeding special diets. Oxidative stress of sperms in different study groups after 28 weeks of feeding special diets studied by** C.** BODIPY probe (%) to analyze the sperm lipid peroxidation, and **D.** dichlorofluorescein diacetate (DCFH-DA) staining (%) to analyze intracellular ROS. Data are expressed as means ± standard error of the mean. HF; High-fat diet, AGEs; Advanced glycation end-products, * ; P<0.05, **; P<0.01, and ***; P<0.001.

### Effects of different diets on sperm DNA damage

As illustrated in Figure 2A, following 28 weeks of keeping the mouse groups on diabetes-inducing diets, the mean percentage of the sperms with damaged DNA was higher in the 45% HF group compared to the control (P=0.07), 60% HF (P=0.02), 60% HF-AGEs (P=0.004), and 45% HF-AGEs (P=0.08) groups.

### Effects of different diets on sperm protamine deficiency

The results of protamine deficiency in different groups are depicted in Figure 2B. The mean of this parameter was significantly higher in the 45% HF (P=0.05) and 60% HF-AGEs (P=0.006) groups compared to the control group.

### Effects of different diets on sperm membrane lipid peroxidation

According to Figure 2C, the mean percentage of lipid peroxidation of the sperm membrane in all the different diet groups experienced an increase in comparison to the control counterpart and the differences of 45% HF (P<0.001), 60% HF (P=0.02), and 45% HF-AGEs (P=0.004) groups was significant. In addition, the mean of this parameter was lower in the 60% HF-AGEs (P=0.07) group in comparison to the 45% HF group, although it was not statistically significant (P>0.05).

### Effects of different diets on sperm intracellular reactive oxygen species

As can be seen in Figure 2D, the means percentage of intracellular ROS of the sperms in four out of five groups with HF and HF-AGEs diets were higher than that in the control group (P<0.001). In contrast, the mean of this parameter was lower in the 60% HF-AGEs group (P=0.07) compared to the 45% HF group, although the difference was not statistically significant. 

## Discussion

AGEs are produced through a non-enzymatic reaction known as glycation/Maillard, using reducing sugars (such as glucose and fructose) in combination with proteins, lipids, or nucleic acids. The AGEs content in the 45% HF and 60% HF diets in our study was not significantly different from the control diet. In contrast, the AGEs content in the 45% HF-AGEs and 60% HF-AGEs were higher than the standard rodent diet due to the high temperature heating process (3).

Fasting blood sugar analysis of the HF and HF-AGEs groups showed increased levels of blood glucose compared to the control group. Interestingly, de Assis et al. (20) reported that HF heat-treated diet has more negative impacts on glucose metabolism and also could induce type 2 diabetes more than the unheated HF diet (i.e. HF-AGE diet). The high glucose level is responsible for hyperglycemia, which is one of the early manifestations of diabetes and could have adverse impacts on semen quality. Hyperglycemia also accelerates AGEs formation, which results in pathophysiological damages in the male reproductive system (5). 

In this research, there were no significant differences among the studied groups in terms of neither sperm concentration nor abnormal sperm morphology. Despite the pathologic conditions of diabetic men, numerous studies have reported that diabetes and obesity may have no direct effects on sperm parameters, but could indirectly impair sperm functions (6, 21-23). 

Unlike the HF-AGEs diets, the obesity-inducing HF diets had significantly negative effects on sperm motility. The HF diet could result in an abnormal level of blood lipid known as dyslipidemia, which could trigger metabolic syndrome and have toxic effects on the reproductive system and semen quality (6, 24, 25). In addition, IR and increased level of glucose following HF diet consumption may alter the level of sperm energy (26). Consequently, both sperm glycolysis and oxidative phosphorylation pathways are disrupted, resulting in impairment of ATP synthesis and abnormal sperm motility. It is also reported that sperm metabolism is negatively influenced by the fatty acid composition of a diet, including high saturated fatty acids and low polyunsaturated fatty acids, leading to the hypothesis that HF diets could cause sperm lipotoxicity (24).

Based on the literature, more deleterious effects on sperm parameters and functions were initially expected with the concomitant rise of saturated fat and AGEs content in the mouse diet. Ironically, instead of showing more reduction of sperm motility in the 60% HF or AGEs group, it was even restored to the control when compared to the 45% HF group. We believe that this discrepancy can be explained by the adaptation mechanism, which is explained in the below paragraphs, although, the literature on this topic is still controversial. An *in vitro* research by Portela et al. (27) suggested that a high concentration of glucose does not affect sperm motility and viability. It is also reported that the disrupted glycolytic process due to the hyperinsulinemia and hyperglycemia in diabetes leads to the decreased uptake of glucose by sperms, which is believed to be associated with an improvement in sperm motility (4).

Due to the fact that glucose and fructose are abundant in germ cells, and sperms are full of polyunsaturated fatty acids, they are prone to glycation reaction and AGEs formation. Chen et al. (28) believe that a diet that is rich in AGEs leads to testicular dysfunction through oxidative stress. It has been shown that AGEs induce ROS formation by inactivating copper, zinc superoxide dismutase (Cu-Zn-SOD), which attenuate cellular antioxidant capacity. AGEs are also very reactive since they act as electron donors and promote superoxide anions formation. On the other hand, oxidative stress is one of the contributory factors in metabolic disorders such as obesity and diabetes, which also accelerates AGEs formation in these physiological conditions. Therefore, there is a valid rationale to assume that there is a feedback loop between AGEs and ROS, which amplifies the formation and biological impacts of each other (4).

Lipid peroxidation is one of the major consequences of AGEs-induced ROS production in cells, which was significantly increased in both HF and HF-AGEs diets compared to the control group. A high level of polyunsaturated fatty acids in the sperm membrane is extremely vulnerable to excess ROS. Karimi et al. (7, 21) have reported that the lipid peroxidation in the semen of diabetic patients is markedly correlated with the high level of AGEs in the semen compared to the non-diabetic individuals. We also analyzed the intracellular ROS, which was significantly higher in the HF and HF-AGEs diet groups than in the control group. Additionally, multiple studies have referred to an inverse relationship between sperm motility and lipid peroxidation (17, 18).

Despite the above explanation on the toxic effects of HF and AGE, both lipid peroxidation and ROS production were high in the 45% HF group compared to the other groups, which were expected to have a more toxic effect. One of the reasons that the percentage of sperm lipid peroxidation, and ROS production were lower while percentage of sperm motility was higher in the 60% HF diet compared to the 45% HF diet could likely be related to the adaptation of mice to 60% diet. In the adaptation stage, the overall situation of blood glucose, insulin level, HOMA-IR, and HOMA beta function are more similar to the control group, therefore, it is not surprising to see better sperm motility, with reducing lipid peroxidation, intracellular ROS production, and reduced DNA damage in the 60% HF vs. 45% HF group.

Despite the results of earlier studies on diabetes that have linked the AGEs augmentation to complications like spermatogenesis impairment, our results showed that although the HF-AGEs diets had higher glucose concentrations and induced the diabetes complications more than the HF diets, they had significantly fewer negative effects on sperm motility, intracellular ROS and lipid peroxidation. Interestingly, Mallidis et al. (22) reported that although semen parameters of diabetic men were not affected, the amount of carboxymethyl-lysine, as the most prominent AGE in the sperm and semen of non-diabetic samples, were considerably higher compared to the diabetic individuals. These observations led to the hypothesis that the deglycation mechanisms would be initiated under the chronic diabetic state, thus eliminating the AGEs. 

Methylglyoxal is known as a precursor of AGEs produced from fructose and glucose following a high sugar intake. Methylglyoxal level is increased in pre-diabetic and diabetic individuals and it leads to disruption of the insulin signaling pathway (29, 30). A recent *in vitro* study by Antognelli et al. (30) demonstrated that an increase in glycolytic flux by spermatogenesis in Sertoli cells leads to methylglyoxal formation as a toxic by-product of glycolysis and it could also elevate the dicarbonyl glycation. They reported that a super-physiological increase in AGEs-induced carbonyl stress, a detoxification mechanism named “glyoxalase pathway,” is activated by Sertoli cells in order to protect the spermatogenesis (30).

In this context, the trend of decreased DNA damage, lipid peroxidation, intracellular ROS production with concomitant improved motility in the 60% HF, 45% HF-AGEs and 60% HG-AGEs diets compared to the 45% HF diet, suggest that an adaptation is taken place following the increased levels of glucose and insulin, which probably activated a detoxification mechanism or the glyoxalase pathway as an anti-glycation defense in the testes and epididymis. In this regard it has been shown that methylglyoxal is converted to D-Lactate, which is less toxic (31). Interestingly, our results also showed that the level of D-Lactate was increased in the latter groups compared to the 45% HF group, indicating that the detoxification mechanism has become activated in these mice. Another potential underlying cause of the milder effect of AGEs on lipid peroxidation is hypoxia. Rodrigues et al. (32) reported that the HF diet does not considerably affect the blood flow in adipose tissue. In contrast, adding methylglyoxal to the HF diet (i.e. HF-AGE diet) induces hypoxia by reducing the blood flow following the glycation in adipose tissue. In other words, with the expansion of adipose tissue and increased vasculature in this tissue, the blood flow to other parts or organs, including testes, is decreased, which results in a state of hypoxia. It is interesting to note that in a 45% HF diet the tissue expansion and weight gain is less than that in the other groups, therefore, the blood flow carrying toxic materials to testes in this group is not reduced compared to the other ones with reduced blood flow (33, 34). Similarly, in our study, both tests showed that the HF-AGEs diets resulted in testicular hypertrophy and hyperplasia compared to the HF groups. Under hypoxia, less oxygen reaches the tissue, when oxygen is necessary for mitochondria to produce energy or ROS (35). Therefore, these mechanisms, in addition to adaptation, may account for reduced ROS and lipid peroxidation in the AGE groups.

Due to the limitations of DNA repair mechanisms in sperms, DNA damage could occur at any step of spermatogenesis, which is a common finding in diabetic patients (21, 36, 37). Although some reports have demonstrated that the AGEs diets exert more damage to DNA than the HF diets (20), our results were in agreement with several studies that concluded the accumulated AGEs in testis, epididymis, and sperm could trigger the protective mechanism of detoxification against AGEs-induced damages in pathological diabetic patients (4, 22, 23). Nevin et al. (4) showed that methylglyoxal, as the most dangerous AGEs in diabetes conditions, did not affect the sperm DNA damage, intracellular ROS, or sperm motility, as the glyoxalase pathway may be involved in the detoxification of AGEs. Additionally, it is reported that soluble AGE receptors (RAGE) is significantly higher in the semen of the infertile men compared to the fertile counterparts (38). Our results indicated the HF-AGEs diets had significantly fewer negative effects than the HF diets and there are moderate differences in DNA damage between the HF and control groups. Similarly, Hu et al. (39) reported that the consumption of the HF foods leads to an increased level of saturated fatty acids in the testes followed by DNA damage and apoptosis in the testes. 

**Fig.3:**
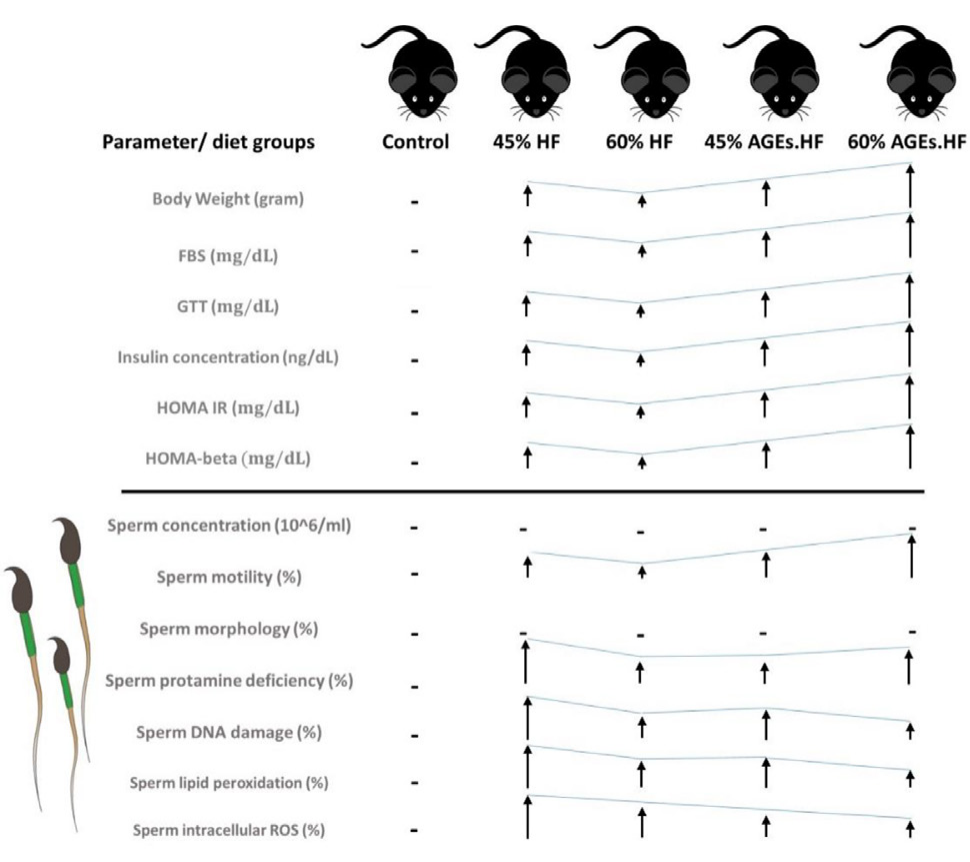
The schematic diagram of experimental results. All groups of diets show an increase in body weight more than the control group diet, although the 60% HF diet is less than the 45% HF, and AGE. HF diets (45% and 60%). Similarity, the assessment of metabolic tests (FBS, GTT, Insulin concentration, HOMA IR, HOMA beta) demonstrate similar results as body weight (g). Unlike sperm motility (%), sperm concentration (10^6^ / ml) and sperm morphology (%) do not show any significant difference among diet groups. While, the assessments of sperm DNA damage (%) showed an increase in 45% HF diet group compared to all the groups while percentage of sperm protamine deficiency demonstrate a highly negative effect in all diet groups compared to control diet group. Approximately, the assessments of sperm ROS [lipid peroxidation (%) and intracellular oxidation (%)] reveal an increase in all the groups compared to control group. HF; High-fat diet, AGE; Advanced glycation end-products, FBS; Fasting blood sugar, GTT; Glucose tolerance test, and HOMA IR; Homeostatic model assessment for insulin resistance

Surprisingly, our results indicated that the 60% HF-AGEs diet induces severe sperm protamine deficiency compared to the control group while it results in a minimum amount of DNA damage in comparison to the HF diets. However, AGEs exert their deleterious effects directly by modifying proteins, lipids, and DNA or indirectly by interacting with their specific receptors in cell surface known as RAGE. In this context, protein carbonylation is the worst consequence of AGEs. ROS also reacts strongly with amino acid residues rich in carbonyl groups like arginine, cysteine, and lysine. In humans, almost 85% of histones are exchanged with protamines during spermatozoa maturation, which is rich in amino acid residues like arginine and cysteine. Other studies have reported that such amino acids, especially arginine, are extremely vulnerable to glyoxal and methylglyoxal that are known as reactive glycating agents (40). Regarding previous findings and the competition of chromomycin A3 (CMA3) dye with protamine for binding to the same sites on DNA, it is possible to believe that toxic AGEs impair disulfide bonds; although their effect did not seem to be strong enough to damage DNA directly. As one of the limitations of our study, the long-term consumption of heated processed foods and the subsequent chronic elevated levels of AGEs in the body may result in adaptation and activation of the protective pathway. Therefore, it was worth investigating the short-term exposure to exogenous AGEs and to compare these results with the effects of longer AGEs intakes. We also did not measure the serum lipids, which should be considered in future research. Additionally, a more thorough understanding of the hidden negative effects of AGEs on DNA conditions requires looking into the genetics and epigenetics in the sperms (Fig .3).

## Conclusion

To sum up, although the sperm concentration, morphology, and morphometric characteristics of the testes were not significantly affected in the C57BL/6 male mice fed with saturated fat- and AGEs-rich diets for 28 weeks, sperm motility, DNA fragmentation, and protamine deficiency as well as membrane and cytoplasmic peroxidation were negatively affected by the HF and HF-AGEs diets. A noteworthy finding in our results was that the adverse effects of the HF diets were more severe than those rich in AGEs, which could be the result of the activation of a protective glyoxalase pathway following the AGEs increase. However, the milder synergistic effect of obesity and diabetes in mice fed by an AGEs-rich diet could mislead our appreciation of the negative hidden effects of these compounds, which demands further studies on the mechanism of action of AGEs and detoxification through the glyoxalase pathway in the body. 
